# The snow must go on: how German cross-country skiers maintained training and performance in the face of COVID-19 lockdowns

**DOI:** 10.3389/fspor.2024.1499738

**Published:** 2024-12-17

**Authors:** H. Kock, A. Schürer, C. A. Staunton, Helen G. Hanstock

**Affiliations:** ^1^ Department of Endurance Sports, Institute for Applied Training Science, Leipzig, Germany; ^2^ Swedish Winter Sports Research Centre, Department of Health Sciences, Mid Sweden University, Östersund, Sweden; ^3^ Department of Environmental and Bioscience, School of Business, Innovation and Sustainability, Halmstad University, Halmstad, Sweden

**Keywords:** endurance training, pandemic, training diaries, training intensity distribution, XC skiing

## Abstract

**Background:**

The Covid-19 pandemic in 2020 led to disruption of sporting events, with athletes obliged to comply with national lockdown restrictions.

**Purpose:**

To investigate the effect of the Covid-19 pandemic restrictions on national-team XC skiers' annual and weekly training distribution from training diaries, results from submaximal and maximal physiological roller ski tests, and competition results from the International Ski and Snowboard Federation (FIS) world cup.

**Methods:**

Annual and weekly training type (specific, non-specific, strength, other) and intensity distribution (TID) data were collected for 12 German XC-skiers (Tier 4/5; BM: 67 ± 7 kg; age 26 ± 3 years; 6♀: V̇O_2max_ 61.3 ± 3.4 ml · kg · min^−1^; 6♂: V̇O_2max_ 72.5 ± 6.2 ml · kg · min^−1^). TID was categorized using a 5-zone scale with Zones 1–2 representative of intensities below the first lactate threshold (LT1), zone 3 between LT1 and LT2, and zones 4–5 above LT2. Training data were grouped by lockdown periods in season 20/21 (L1/L2) and compared to data from the corresponding weeks in 19/20 (C1/C2). Laboratory testing was performed in the general preparation period prior to competition for both seasons. Differences between seasons (C1/C2 vs. L1/L2) in training and performance variables were analysed using repeated-measures ANOVA and linear mixed models.

**Results:**

Total annual training duration increased by 9% during 20/21 (928 ± 79 h · year^−1^) compared to 19/20 (852 ± 73 h · year^−1^). During L1, skiers achieved a greater weekly training duration (mean differences (Δ*x¯*: 7.7 h · week^−1^) compared to C1, due to an increase in non-specific training (Δ*x¯*: 7.0 h · week^−1^), whereas L2 resulted in greater weekly training compared with C2 due to a higher specific endurance training volume (Δ*x¯*: 1.4 h · week^−1^). In 20/21 skiers performed a higher volume of Zone 1 (Δ*x¯*: 149 h · year^−1^). Laboratory test- and FIS racing performance improved from 19/20 to 20/21.

**Conclusion:**

German XC skiers' training characteristics, laboratory- and racing performance were significantly different between the two seasons. In fact, training duration as well as laboratory- and racing performance increased from 19/20 to 20/21. In spite of seasonal variation in performance and training within an Olympic cycle these findings might suggest that skiers adapted their training effectively to pandemic constraints, ultimately enhancing performance outcomes.

## Introduction

Due to intense physiological and technical demands over undulating terrain, cross-country (XC) skiing is among the most demanding endurance sports (1, 2). Elite XC skiers have exhibited some of the highest maximal oxygen uptake values reaching upwards of >70 and >80 ml · min^−1^ · kg^−1^ for women and men, respectively (3). To develop and use such a high aerobic capacity, XC skiers train around 750–1,000 hours per year, distributed across 400–500 sessions annually, using training types such as on-snow skiing, roller skiing, running, cycling and strength training (4, 5). Approximately 90% of XC skiers' total annual training duration consists of endurance training, with the remaining 10% comprised of strength and sprint training (1, 4, 5). The majority of XC skiers' annual training duration (∼60%) takes place during the general preparation period (GPP) with the remaining training (∼40%) completed during the specific preparation (SP) and competition phases (CP) (4, 5). During the GPP, XC skiers usually undertake a high proportion of low-intensity training, comprised of 50%–60% sport-specific endurance training and the remainder non-specific training. Towards the SP phase and the CP, the total training duration decreases whereas the duration of high-intensity training (including competitions) and sport-specific activities increases (2).

The outbreak of the Covid-19 pandemic in late December 2019 and its subsequent rapid international spread led to increasing national and international regulations to contain the disease (6). Both minor stressors and major life events can disrupt individuals' daily routines and are known to influence physical activity behaviour (7). The pandemic was unique in that societal lockdown restrictions limited access to facilities and opportunities for many types of physical activity. In the context of stressful life events, the pandemic was unusual, since often it is not the event itself that drives physical activity-related behaviour change, but rather the daily stressors and challenges they create in a person's life (8). During the pandemic, negative psychosocial effects such as heightened anxiety and stress (8) may have been further exacerbated by a lack of social connections, which usually help to maintain general well-being (9). Germany, for example, had some of the harshest public restrictions in the world, including social distancing, movement restrictions, and closure of most public areas, including training facilities (10). Only individual outdoor training, in close proximity to one's home and without direct contact with others, was permitted during the first German lockdown (11). On the other hand, the pandemic also gave rise to digital training tools, either through online software solutions coupled with smart trainers (e.g., ski ergometers or cycle trainers) or through digital platforms in conjunction with GPS monitoring devices to provide virtual racing experiences for events that were otherwise cancelled (12). Such solutions might have mitigated the effects of restrictions on racing and training opportunities (13). Ultimately, the pandemic had a profound impact on both elite and recreational sport due to effects both at the individual and societal levels (14). As a social phenomenon, it affected a diverse range of individuals and groups, including those who actively participate in sport as well as those who consume, shape and report it.

Several studies have evaluated the effects of the Covid-19 pandemic on the training of professional athletes from >150 countries, although the majority of these focused on team sports. An overall reduction in training volume, specificity, intensity, frequency and duration during lockdowns, despite an increase in home-based and solo training, has been reported in multiple studies (11, 15–23). Generally, these studies indicate a decrease in exercise capacity and competition performance due to pandemic restrictions. However, it seems that higher-level athletes coped better with restrictions than lower-level athletes (23), potentially due to having better established routines for training or better financial support, both of which could have also been positive for athletes' motivation and mental wellbeing (24, 25).

In the case of German XC skiers, the pandemic led to an early conclusion to the 2019/20 international competition season at the beginning of March 2020, leaving XC skiers in a state of general uncertainty regarding future competition and training opportunities relevant for team selections and subsequent financial support. Due to the lockdown restrictions, XC skiers and coaches were likely forced to alter their training plan. German athletes were potentially more affected than their Nordic peers who were generally subjected to lighter restrictions, based on a stringency index of pandemic policies (26). If XC skiers' training quantity was affected during lockdowns, it is likely that this would be represented not only by training diary data but even in the available physiological and performance data, as adaptations to training are likely to be compromised if a sufficient training stimulus is not available (27, 28). However, not only the training quantity is important for athletic success but also the training quality expressed as the how and why training practices are performed (29, 30). Thus, even if athletes had sufficient training quality, missing out on certain contextual variables, such as goal setting or in-person interactions with coaches and peers for feedback and support, could mean that athletic success is not guaranteed (30).

To date, no studies have evaluated the effect of the Covid-19 pandemic on the quantitative differences in executed training over the pre and post pandemic periods and performance capacity of national-team XC skiers. This study therefore aimed to investigate the effect of the German Covid-19 pandemic restrictions on national-team XC skiers' (a) annual and weekly training distribution from training diaries, (b) physiological parameters obtained from laboratory testing and (c) competition results in the 20/21 season compared to the 19/20 season.

## Materials and methods

### Participants

Twelve Tier 4–5 (31) XC skiers participated in this retrospective study. Skiers comprised 6 female (age: 25 ± 1 years; body mass: 62 ± 7 kg; body height: 171 ± 6 cm; V̇O_2max_: 3.8 ± 0.4 L · min^−1^/61.3 ± 3.4 ml · kg · min^−1^) and 6 male (age: 27 ± 3 years; body mass: 73 ± 3 kg; body height: 180 ± 4 cm; V̇O_2max_: 5.3 ± 0.4 L · min^−1^/72.5 ± 6.2 ml · kg · min^−1^) athletes. All participants were living in Germany during the time of data collection, competing on the FIS XC World Cup during the period of data collection. All participants provided *a priori* written informed consent to use their training and physiological test data for research. The team medical doctor confirmed that none of the athletes reported a Covid-19 infection throughout the observation period. The study was approved by the Institutional Review Board of the Institute for Applied Training Sciences (approval number: ER_2022.16.03_10).

### Background

During the 2019/20 season (19/20), XC skiers trained normally until the premature end of the FIS racing season. Upon returning from the cancelled FIS final World Cup events in Canada, German athletes were obliged to adhere to restrictions announced including only movement in close proximity to one's home, mask mandates in public indoor spaces as well as controlled access to public and sporting facilities based on vaccination/infection status by the German Federal Government on 23/03/2020 (10). This phase will be referred to as Lockdown 1 (L1), with a matched control period during the corresponding calendar weeks in 2019/20, denoted as Control 1 (C1; Table 1). A second lockdown with lighter restrictions was declared on 02/11/2020. This phase will be referred to as Lockdown 2 (L2) and its control period during the corresponding calendar weeks in 2019/20 as Control 2 (C2). The 20/21 competitive season proceeded as planned, with athletes and support personnel subject to increase illness surveillance strategies including mask mandates at events, frequent testing and strict self-isolation following a positive test, described in detail elsewhere (33, 34).

**Table 1:** Training zones used to classify training intensity distribution, adapted from Seiler (32).

Intensity	Heart Rate	V̇O_2_	Lactate
[Zone]	[% HR_max_]	[%V̇O_2max_]	[mmol · L^−1^]
Zone 1	60–72	50–65	<1.5
Zone 2	73–82	66–80	1.5–2.5
Zone 3	83–87	81–87	2.5–4.0
Zone 4	88–92	88–93	3.0–5.0
Zone 5	93–100	94–100	5.0–10.0

### Training data

Data were obtained retrospectively from skiers' training diaries. During the study period, skiers documented their training through a web-based training diary (TDSKI) on the day of each workout. A cumulative export of all recorded data was performed from TDSKI. The training data includes training duration and training intensity distribution based on heart rate time-in-zone data as well as training type (on-snow skiing, roller skiing, running, cycling, strength training, other training). Endurance training variables were further categorised as specific endurance training (on-snow skiing, roller skiing) and non-specific endurance training (running, cycling, canoeing, etc.). Athletes documented their specific endurance- and running training based upon a 5-zone heart rate-based intensity scale adapted from (32) (Table 1). Athletes could modify their reported zone based upon field lactate measures (i.e., if HR was in zone 4 but lactate was in zone 3, the athletes would report zone 3). Accordingly, zone 1 and 2 represent intensities below the first lactate threshold (LT1), zone 3 between LT1 and LT2, and zones 4–5 above LT2. Subsequently, training data were grouped by season and according to the lockdown periods across a season by using a weekly average approach (Table 2).

**Table 2:** Study timeline and periods for the 19/20 and 20/21 competition seasons.

Period	Weeks	Equivalent month/Start-end dates
Transition Period (TP)	14–17	Apr
General Preparation Period 1 (GP1)	18–30	Apr–Jul
General Preparation Period 2 (GP2)	31–39	Jul–Sep
Specific Preparation Period (SP)	40–48	Oct–Nov
Competition Period (CP)	49–13	Dec–Mar
Lockdown 1 (L1)^1^	13–19	23/03/2020–10/05/2020
Lockdown 2 (L2)^1^	45–8	02/11/2020–28/02/2021

^1^ Lockdown periods occurred only in 20/21; the corresponding periods in the 19/20 season are referred to as C1 and C2.

### Physiological characteristics

Physiological laboratory tests were performed at the end of skiers' first GP period (GP1) in July (week 30) and at the end of their second GP period (GP2) in September (week 39) and the data obtained retrospectively. XC skiers completed a 5-stage incremental submaximal test on a 2-degree incline using the one-skate (gear 3 or V2) skating technique exclusively. Each stage corresponded to an intensity zone (Table 1), starting at zone 1 and progressing every 6 min to the next zone. A 2-min rest period between stages allowed for capillary blood lactate sampling. Starting velocities were set at fixed speeds (♀ = 2.9 (+0.6) m/s; ♂ = 3.2 (+0.7) m/s), but skiers could adjust treadmill speed using a tension cord around their hips. This setup let them control their speed to match their heart rate displayed on the treadmill monitor, maintaining the intensity range specified for each level. After the incremental test all athletes performed an active recovery of 8 min before a ramp protocol to exhaustion was performed, with athletes being able to self-select sub techniques. During the ramp test athletes complete three consecutive 20-s intervals at inclines of 1, 4, and 7 degrees per stage with increasing velocities per stage (♀ = 0.22, 0.18, 0.13 m/s; ♂ = 0.25, 0.20, 0.15 m/s). Both tests were performed on roller-skis [SRB SR01, Medium, NNN-Bindings (Rottefella, Lierstranda, Norway)] on a broad treadmill with 3,000 × 4,500 mm dimensions (Poma-Porschendorf, Germany). Capillary blood samples were obtained from the ear lobe and blood lactate (BLa) analysed using a point-of-care device (SUPER GL, Dr. Müller Gerätebau GmbH, Freital, Germany).

Velocity at a fixed lactate value of 3 mmol · L^−1^ (vBLa3) was determined using WinLactat 5 software (Mesics, Münster, Germany). Ventilation and gas exchange analyses were performed using a breath-by-breath system (Metalyzer3B, Cortex, Leipzig, Germany) in order to determine V̇O_2max_, defined as the highest 21-breath moving average of V̇O_2_ (35). Maximal heart rate (HR_max_) was defined as the highest HR-value reached during the ramp protocol. Test duration is defined as the maximal achieved time and stages as the total number of completed stages during the ramp protocol.

### Racing performance

Racing results were obtained from the official website of the FIS (https://www.fis-ski.com/DB/cross-country/calendar-results.html). According to the FIS point system, points can be calculated for each athlete competing in a specific race. Results for each athlete were obtained from the 8th List of each season which takes the average of the best five results over a 12-month period. FIS points for each individual race and participant were calculated according to the international rules described in detail by (36).

### Statistical analysis

Statistical analyses were performed using IBM SPSS Statistics (Version 27.0; IBM Corporation, NY) with level of significance set at *α* < 0.05. The data were checked for normality using the Shapiro–Wilk test and visual inspection of *Q*–*Q*-plots confirmed that the assumption of normality was not violated. Group data were expressed as mean ± standard deviation (SD). Homogeneity of variance was assessed using Levene's test. Training data from male and female athletes were pooled for analysis, as independent *t*-tests revealed no significant differences between sexes for all training variables. Repeated measures two-way ANOVAs (within-subjects factors: year; were performed to identify whether year (19/20; 20/21) and/or exercise type (Specific; Non-Specific; Strength; Other) influenced the pattern of training demands over the training year. Repeated measures two-way ANOVAs were performed to examine the effect of year (19/20; 20/21) and/or exercise type (specific endurance training; non-specific endurance training; strength; other) on the training performed within each lockdown period. Repeated measures one-way ANOVAs (within-subjects factor: year) were used to identify whether year (19/20; 20/21) influenced the pattern of training intensity distribution (Zone 1–Zone 5) for each lockdown period or for the entire training year. A two-way ANOVA was performed to determine the effect of year (19/20; 20/21) and test week (30; 39) on laboratory test variables. A paired samples *t*-test was performed to determine the effect of year (19/20; 20/21) on ski performance (FIS points). For all ANOVAs, effect sizes are presented as partial eta-squared statistic (*η*^2^*_p_*) or Cohen's *d*. Significant interactions were followed up with simple main effect analyses with pairwise comparisons using Bonferroni corrections; differences between means for paired comparisons are reported as Δ*x¯*. Greenhouse-Geiser corrections were used if the assumption of sphericity was violated.

## Results

### Training characteristics

**Figure 1:**
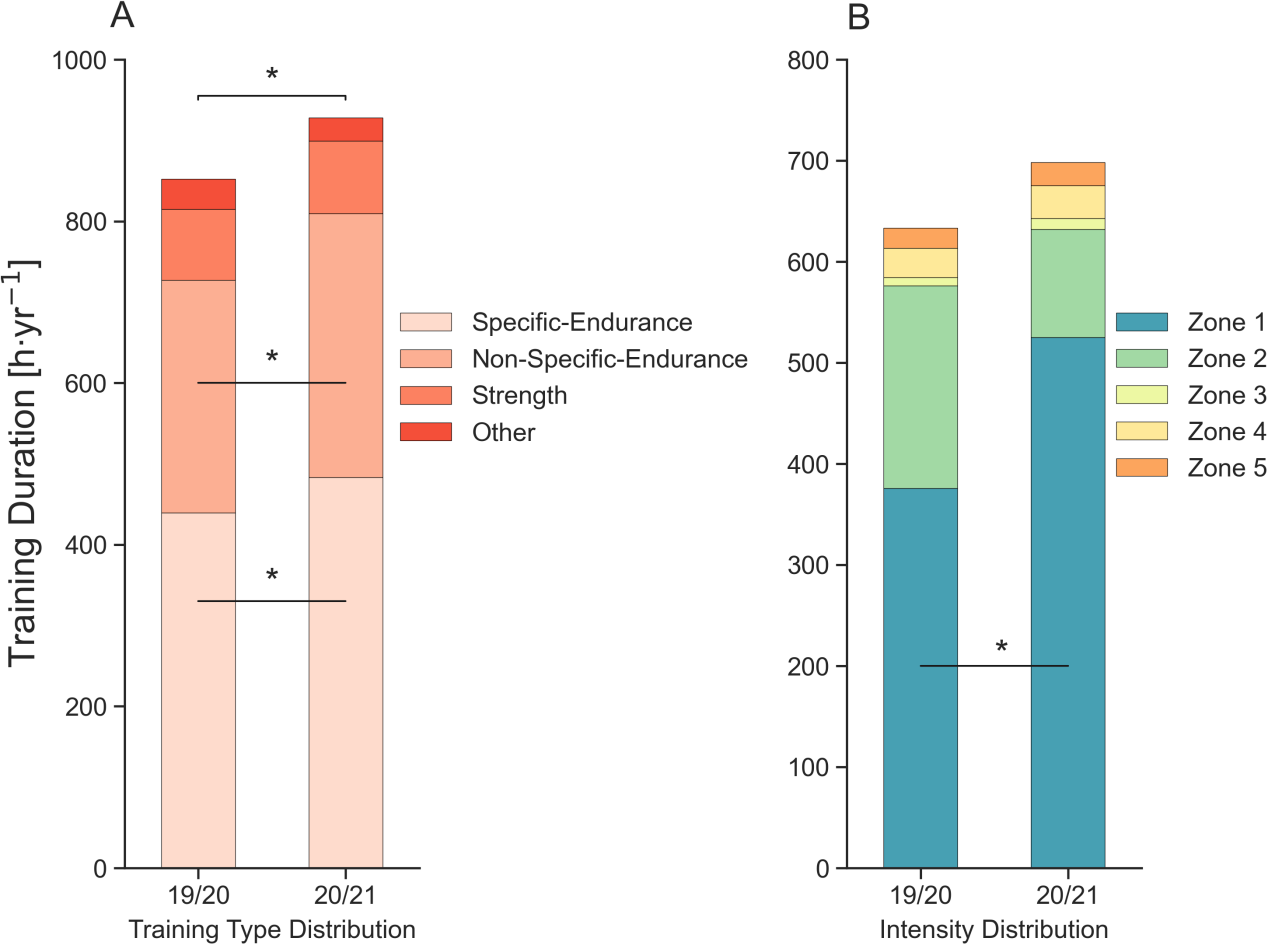
Training characteristics across season and period, *P = <0.05. **(A)** Annual training type distribution; **(B)** annual training intensity distribution for specific training types and running.

Figure 1 displays the training types and intensities across both seasons and periods, and further information about the statistical interactions is provided in Supplementary Table 2. A training type × training season interaction (*F*_3,33_ = 7.805, *P* = 0.03, *η*^2^*_p_* = 0.415) revealed that total annual training duration increased from 852 ± 73 h · year^−1^ in 19/20 to 928 ± 79 h · year^−1^ in 20/21. Specific- and non-specific endurance training duration accounted for 52% and 35% of total training, with the remaining 10% comprised of strength training and 3% of other training types (Figure 1A). Specific endurance training duration increased from 440 ± 70 h · year^−1^ to 483 ± 58 h · year^−1^ and non-specific endurance training duration also increased from 288 ± 36 h · year^−1^ to 326 ± 46 h · year^−1^. There was no difference in the amount of strength training or other training between 19/20 and 20/21.

The majority of training duration (91%) was spent below LT1, ∼1% between LT1-LT2 and the remaining ∼8% above LT2 during the 19/20 and 20/21 seasons (Figure 1). There was a training intensity distribution × training season interaction (*F*_4,44_ = 4.877, *P* = 0.048, *η*^2^*_p_* = 0.307); XC skiers completed more zone 1 training in 20/21 (525 ± 156 h · year^−1^) compared to 19/20 (376 ± 133 h · year^−1^). There were no significant changes between other zones (Figure 1A).

**Figure 2:**
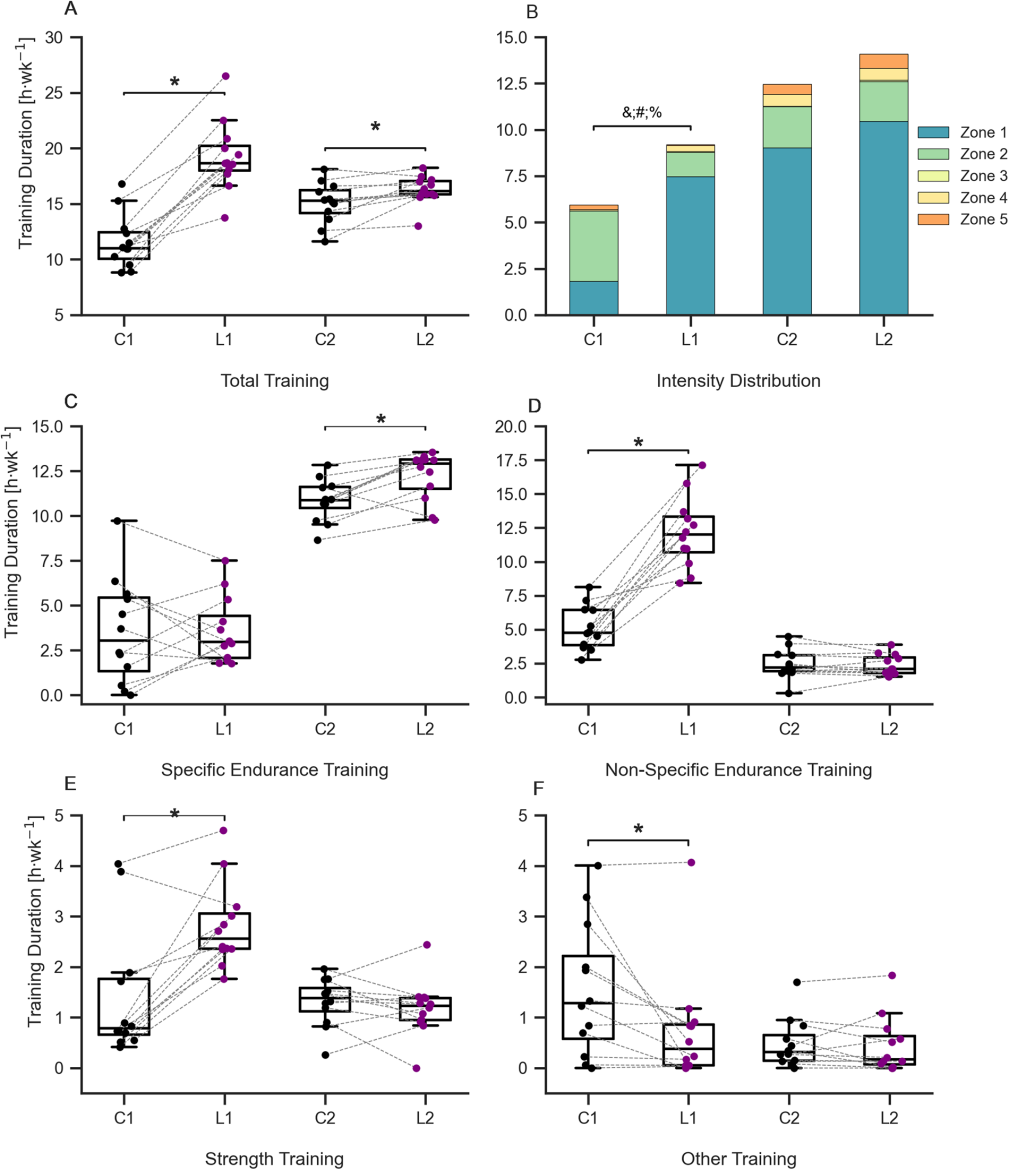
Training characteristics across lockdowns (L1, L2) and control periods (C1, C2), *P < 0.05 vs. equivalent control period. The specific colours in subplots A, C, D and F refer to each specific period: C1/C2, black; L1/L2, purple. **(A)** Total weekly training duration, **(B)** training intensity distribution, &, difference between Z1; %, difference between Z4; #, difference Z5 **(C)** weekly duration of specific endurance training **(D)** weekly duration of non-specific endurance training; **(E)** weekly duration of strength training **(F)** weekly duration of other training.

Figure 2 displays the training characteristics between the lockdown and control periods. There was a training type × lockdown period interaction (*F*_3,33_ = 26.774, *P* < 0.001, *η*^2^*_p_* = 0.709); skiers performed a greater total training duration in L1 compared to C1 (Δ*x¯*: 7.7 h · week^−1^) as well as a greater total training duration in L2 compared to C2 (Δ*x¯*: 1.2 h · week^−1^; Figure 2A). Skiers performed more zone 1 (Δ*x¯*: 5.7 h · week^−1^) and zone 4 training (Δ*x¯*: 0.3 h · week^−1^) but less zone 5 training (Δ*x¯*: 0.2 h · week^−1^) during L1 compared to C1 (Figure 2A). There was also a trend toward less zone 2 training (Δ*x¯*: 2.5 h · week^−1^, *P* = 0.055) during L1 compared to C1. There were also differences in modality between control periods and lockdowns. Specific endurance training duration was greater in L2 compared to C2 (Figure 2C), whereas non-specific endurance training was greater in L1 compared to C1 (Figure 2D). Further analyses revealed an increase in strength training (Figure 2E) and a decrease in other training (Figure 2F) during L1 compared to C1.

### Laboratory test data

Laboratory performance data from both seasons are presented in Table 3. Significant main effects of season were found for V̇O_2max_, v_max_, vBLa3, stage and test duration, indicating that skiers had greater performance capacity in 20/21 compared to 19/20. Further significant main effects of week were found for HR_max_, v_max_, stage, vBLa3, and test duration, indicating that skiers had greater performance capacity in week 39 compared to week 30.

**Table 3:** Laboratory performance variables between week (30/39) and season without (19/20) and with (20/21) COVID-19 lockdowns.data are presented as mean ± SD. v_max_ represents the highest velocity achieved in the ramp test. vBLa3 corresponds to the velocity at a fixed lactate threshold of 3 mmol · L^−1^ determined during the incremental test.

Variable	19/20		20/21		*P*	*P*
	W30	W39	W30	W39	Season	Week
V̇O_2max_ [L · min^−1^]	4.45 ± 0.97	4.26 ± 0.98	4.56 ± 0.85	4.6 ± 0.84	0.037	
HR_max_ [beats · min^−1^]	186 ± 5	188 ± 6	185 ± 6	188 ± 6		0.011
v_max_ [m · s^−1^]	5.17 ± 0.57	5.32 ± 0.49	5.33 ± 0.46	5.42 ± 0.44	0.041	0.022
vBLa3 [m · s^−1^]	4.57 ± 0.36	4.76 ± 0.46	4.89 ± 0.38	5.01 ± 0.4	0.013	0.034
Stages	7.56 ± 1.74	8.2 ± 1.40	8.25 ± 1.14	8.91 ± 0.94	0.042	0.025
Test duration [min]	6.88 ± 1.79	7.54 ± 1.37	7.47 ± 1.06	8.22 ± 0.9	0.048	0.02

### Racing performance

FIS results from both seasons are presented in Table 4. Athletes performed significantly better in the distance and sprint events in the competition season 20/21, indicated by lower FIS-points during 20/21 compared to 19/20.

**Table 4:** Ski racing performance variables during the competition seasons without (19/20) and with (20/21) COVID-19 lockdowns. Data are retrieved from the International Ski and Snowboard Federation (FIS) 8th list and presented as mean ± SD.

Variable	19/20	20/21	*P*
			Season
FIS Distance points	38.77 ± 13.97	32.38 ± 16	0.001
FIS Sprint points	100.34 ± 53.49	83.79 ± 44.51	0.002

## Discussion

To our knowledge, this study is the first to report training and performance data from national-team XC skiers during the Covid-19 pandemic, and is also the first to report annual training characteristics among German XC skiers. The main findings of this study were: (1) skiers’ annual training duration increased by approximately 9% during the Covid-19 impacted year, with the majority of the increase accounted for by an increase in the duration of low-intensity (zone 1) training; (2) the first lockdown impacted training, where total duration was greater compared to the control period. This was mostly explained by increases in the duration of non-specific endurance and strength training. On the other hand, the second lockdown had little impact on training, where athletes trained in a manner similar to the preceding year; and (3) XC skiers improved upon their laboratory performance variables and FIS-points from the first to the second year, in spite of the pandemic.

Studies from a variety of other sports, including cycling and sprint canoeing, have observed that Covid-19 lockdown restrictions reduced training duration among elite- and junior athletes (11, 18). Several endurance training studies have previously observed that a high training duration is a prerequisite for successful endurance performance (1, 2, 4, 37–39). As such, reductions in training duration might jeopardise athletic performance development (11, 18, 23). However, we found no evidence of an attenuation in progression of annual training duration in our cohort of German national team XC skiers during the first year of the Covid-19 pandemic. On the contrary, there was a positive trend in total training duration that was mostly explained by an increase in low-intensity (zone 1) training. Athletes from this study trained 852 ± 73 h · year^−1^ in 2019/20 and were able to further progress by approximately 9% to 928 ± 79 h · year^−1^ in 2020/21. These annual training durations are within the range of annual training durations previously reported in elite level XC skiing athletes (2, 4, 39, 40). The increase in total training duration by 9% between seasons is also in line with the ∼5%–10% annual increase previously reported among junior and senior XC skiers (39, 41–44). In addition, the training intensity distribution from this study followed a polarised model, where the large majority of training was performed as low-intensity (∼85%). These findings are in line with previous research that observed from elite endurance athletes, that the majority (88%–91%) of training duration is performed with an intensity below LT1 (4, 37, 38, 45).

During both lockdowns, skiers increased their training duration compared to the control periods in the previous year. This finding contrasts the reported effects of lockdowns on the training duration of elite Spanish cyclists and German sprint canoeists where initial Covid-19 lockdown(s) reduced weekly training duration by 34% and 28%, respectively (11, 18). However, these studies compared lockdown training durations to the weeks prior within the same year and not to corresponding periods during the previous year. As such, it is difficult to determine whether changes in training performed were related to planned periodisation or Covid-19 restrictions. A strength of the present study is that data were collected and compared during the same phase within the periodised training program, although this matching might still have been affected by the cancellation of late-season races following the outbreak of Covid-19, effectively shifting the periodisation forward for the following year.

During L1, training duration increased primarily due to an increase in low-intensity, non-specific endurance training and strength training. In general, L1 occurred toward the end of the CP and spanned the TP and part of the GP1 under normal circumstances for XC skiers' training. Therefore, we could expect that skiers' total training duration would be initially reduced post CP and progressively increased through GP1, usually focusing on performing high volumes of LIT utilizing a large proportion of non-specific training (4, 40, 46). The initial decrease in total training duration was more accentuated in C1 compared to L1. These changes in skiers' training characteristics during L1 might be attributed to: (1) methodical changes in the training plan, (2) training periodisation being shifted forward in time ahead of the 20/21 season, due to the early conclusion of the previous season; and/or (3) specific pandemic coping strategies such as training alone, primarily outdoors or at home utilising online training solutions, and a lack of group training and training camps to mitigate infection risk, while studies from the general population generally show trends of higher stress levels and poorer mental health during the early phases of the pandemic (47), which may have decreased athletes' motivation and availability to train, a minority of individuals reported less stress, perhaps due to a reduction of total demands. For example, reduced stress during the early lockdowns in a Swiss population was associated with higher levels of physical activity during the early stage of the pandemic (48). Although we have no data on athletes' stress and wellbeing *per se*, it is possible that the participating XC skiers could have fallen within the latter group, if the demands for travel and competition were reduced. An absence of illnesses due to social and physical distancing may have led to increased training availability, which is a prerequisite for athletic success both in cross-country skiers and other endurance sports (49, 50). Moreover, these athletes may have been particularly motivated to train effectively during this period, since their performance in the upcoming season would determine Olympic Winter Games selection. Based on these findings, we suggest that Covid-19 pandemic-induced restrictions did not attenuate the duration of training in elite XC skiers', but rather that athletes were able to continue training and improve their performance capacity in the year following implementation of Covid-19 restrictions. This is in contrast to previous findings in other sports, which indicate that the weekly training duration across different intensity zones was significantly reduced by 26%–53% (LIT-HIT) during initial lockdowns, although mostly for summer or team sports (23).

During L2, skiers increased their total training duration via an increase in specific training, with no differences in training intensity distribution between seasons. In general, L2 encompasses parts of the SP and CP. During these phases the HIT will be usually increased for competition preparatory purposes as well as due to an increasing number of competitions. In tandem, the majority of endurance training shifts towards specific training types, with non-specific training types aiding as compensatory training (4, 40, 46). The increase in specific training duration in L2 compared to C2 shows that, despite lighter lockdown restrictions during L2, athletes were not limited in their ability to training according to seasonal norms. Possible explanations include weather conditions that provided snow for on-snow skiing training and/or that bubble strategies enabled international competitions to resume, with athletes and team staff required to take frequent PCR testing before and after each competition as well as the implementation of mask mandates [explained in detail by (51)]. As a result, athletes had the opportunity to train and/or compete on skis as in the previous season (33). In addition, the German Skiing Federation implemented over 100 on-snow events across the country, which provided over 6,000 athletes with the opportunity to participate in ski racing during the competition period in 2020/21 (November–March). The timing results were retrieved from GPS watches or manually timed by each participant and centrally compiled to produce “race” results for each age group. However, we do not know whether the surveyed athletes engaged specifically in this initiative. While training at competition-like speeds has been shown to be important in XC skiers' training (52), in our data it does not seem that athletes lost valuable training at competition speed on snow, performed under training or racing conditions, nor exposure to racing against international competitors, as a result of L2.

Furthermore, significant improvements in participants' laboratory performance variables were indictive of positive adaptations between seasons in V̇O_2max,_ v_max_, vBLa3, test stages completed and test duration. In addition, some of the variables (HR_max_, v_max_, stage, vBLa3_,_ test duration) also improved from week 30–39. Studies reporting changes in performance variables following lockdown restrictions are scarce in endurance sports. However, performance variables in elite cyclists declined after lockdown restrictions by 9%–12% (18). In contrast, athletes in the present study not only improved their laboratory performance, but were also able to improve their racing performance in the distance and sprint events from 19/20 to 20/21 as demonstrated by improvements in FIS points (Table 3). While performance improvements are typically anticipated over an Olympic cycle leading up to the Olympic season, these advancements may not necessarily outpace those of fellow athletes striving to reach their peak during the Olympic Winter Games (39).

To summarise, it seems clear that the coping strategies employed by athletes and coaches through the German lockdowns in the present study did not impact their ability to train effectively according to seasonal norms within their sport. This concurs with findings by Washif et al. (23) that athletes of higher performance levels were broadly able to maintain their training routines during lockdowns compared to normal conditions. Achieving a high training quality within the integrated training process of preparation, execution and debriefing in close connection with a coach might have been key in the development of the XC skiers from this study (29). Frequent communication strategies, especially focusing on important training sessions were used within the national team structure (personal communication A. Schürer) which has been shown to positively affect athletes' coping strategies throughout stressful life events and results in an overall greater engagement in the training process as well as a positive effect on mental health, which might otherwise have adversely affected their training commitment and overall life satisfaction (24, 53). Such communication, complemented by structured monitoring tools and controlled testing protocols, was essential in supporting athletes' abilities to not only sustain but improve performance levels despite training limitations. Most of the XC skiers in the present study had financial support from federal structures (e.g., army, police), potentially mitigating negative financial concerns associated with anxiety, depression, and reduced life satisfaction (24). The contrasting effects of the first and second lockdowns on training patterns demonstrate the flexibility of training approaches under varying levels of restrictions. Focusing on low-intensity work as well as on strength training in endurance sports suggests that in times of disruption, maintaining a substantial base of low intensity stimuli can provide stability in athletic conditioning, potentially aiding performance progression. This might also underscore that continuity in training practices throughout challenging periods to mitigate detraining effects might facilitate seamless transitions into subsequent, more demanding training phases. whenever restrictions are lifted.

In light of the findings presented in this study, it is evident that further research is required to gain a deeper understanding of the efficacy of adaptive strategies to maintain sports performance during challenging periods for athletes and their support systems. Studies such as this on the Covid-19 pandemic offer some context-specific insights, but could also inform preparedness for universal, regional or local challenges, such as future pandemics, geopolitical instability, natural disasters, or loss of access to coaching, facilities or funding. The implementation of comprehensive and personalised monitoring systems that account for both physical and cognitive load, in conjunction with regular assessments through laboratory testing or benchmark workouts, can facilitate the acquisition of actionable insights into athletes’ progress, thereby informing the implementation of tailored training programming (54). This approach not only supports physical performance but also fosters mental resilience by addressing stressors collectively and highlights the importance of adaptability, robust support networks, and intentional training processes in the pursuit of peak performance (55). These “lessons learned” from the present study are summarised in Table 5.

**Table 5:** Lessons learned from the present study on how cross-country skiers maintained training and performance in the face of COVID-19 lockdowns.

Key lesson		Details
Keep training plans flexible	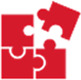	Maintaining flexibility in the training plan can help athletes and their teams to cope with and adapt to unplanned disruptions
Maintain an endurance base	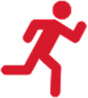	There are multiple options to maintain low-intensity training, both specific and non-specific to XC skiing.
Communicate clearly and consistently	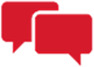	Transparent communication can help athletes to cope with disruptions and maintain training and wellbeing.
Ensure stable institutional and financial support	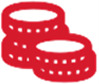	Structural support is important to maintain conditions for training and wellbeing.
Use personalised monitoring for better insights	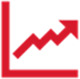	Systems that allow reporting of objective and subjective measures provide valuable and actionable insight into athletes’ progress.
Conduct regular testing for progress feedback	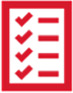	Regular standardised testing either in the laboratory or through benchmark sessions provides objective feedback on athletes’ progress.

### Limitations

The retrospective study design is a limitation since the research team were unable to employ quality control measures at the time of recording. Accordingly, the accuracy of self-reported training data can be questioned since athletes may have tendencies to over- or under-report specific variables, such as intensity, based on training experience and/or tolerance (56). In retrospect, information about athletes' psychological wellbeing could have given further insights into their specific coping strategies, but no information of this character was available within the present dataset. Athletes who participated in the present study were geographically dispersed within Germany and their different coaches laid out individual training plans. As retrospective access to planned training was not available, this study utilised only reported training, rather than reported vs. planned training, hence changes in the observed differences remain speculative regarding restrictions induced changes or general changes in the decision-making of the training process. In addition, several factors may influence the validity of comparing FIS points and rankings across seasons, including differences in weather and track conditions, ski preparation, participating nations, and, especially during the pandemic, travel restrictions, mandatory testing and quarantines potentially leading to reduced participation in some events during the Covid-19 season. Finally, while recruitment was successful, this study had low statistical power by nature of the limited pool of elite-level XC skiing athletes in Germany.

## Conclusion

The annual training duration of XC skiers increased by approximately 9% during the Covid-19 impacted year, with the majority of the increase accounted for by an increase in the duration of low-intensity (zone 1) training. The first lockdown positively impacted training, resulting in a greater total training duration compared to the same period within the preceding year. This was mostly explained by increases in the duration non-specific endurance and strength training. On the other hand, the second lockdown had little impact on training, and athletes trained in a manner similar to the preceding year. In tandem, skiers improved their laboratory and racing performance from the 19/20 to the 20/21 season. These findings suggest that Covid-19 restrictions had little quantitative impact on elite level XC skiers' training and performance. To maintain good training quality we encourage frequent, purposeful communication between coaches and athletes within the training process, and among athletes themselves. This communication can serve as a crucial coping strategy, potentially contributing to the fulfilment of planned training quantities and subsequent performance enhancements. Furthermore, evaluations of training progress through laboratory testing or the analysis of benchmark workouts may be a reasonable method of maintaining motivation among athletes.

## Data Availability

The raw data supporting the conclusions of this article will be made available by the authors upon reasonable request.
