# Supplementary material for: The snow must go on: how German cross-country skiers maintained training and performance in the face of COVID-19 lockdowns
Source: Front Sports Act Living. 2024 Dec 17;6:1499738. doi: 10.3389/fspor.2024.1499738 (PMC11685230; doi:10.3389/fspor.2024.1499738)
Supplement: Supplementary file 1 [file Table1.docx]

**Supplementary Table 1**: Annual training patterns of German XC skiers across the seasons
19/20 and 20/21. Data are mean ± SD. *P* value and effect size (ES) statistics represent
pairwise comparisons between respective seasons (C1 vs. L1, C2 vs. L2).

| **Annual training patterns** | **Season** | | | | | | ***P*** | **ES** |
| --- | --- | --- | --- | --- | --- | --- | --- | --- |
|  | 19/20 | | | 20/21 | | |  | |
|  |  | | |  | | |  |  |
| Training Duration [h·y^-1^] | 852.12 | ± | 73.24 | 927.94 | ± | 78.99 | **< 0.001** | **0.85** |
| **Training Mode:** |  |  |  |  |  |  |  |  |
| Specific [h·y^-1^] | 439.55 | ± | 69.94 | 483.19 | ± | 58.14 | **0.003** | **0.56** |
| Non-specific [h·y^-1^] | 412.57 | ± | 65.56 | 444.75 | ± | 64.40 | **0.001** | **0.63** |
| Strength [h·y^-1^] | 87.40 | ± | 19.60 | 89.85 | ± | 19.30 | 0.705 | 0.01 |
| Other [h·y^-1^] | 37.23 | ± | 23.89 | 28.54 | ± | 32.22 | 0.136 | 0.19 |
| **Intensity Distribution:** |  |  |  |  |  |  |  |  |
| Zone 1 [h·y^-1^] | 375.74 | ± | 133.48 | 524.98 | ± | 156.47 | **0.021** | **0.40** |
| Zone 2 [h·y^-1^] | 200.51 | ± | 147.18 | 106.86 | ± | 170.67 | 0.123 | 0.20 |
| Zone 3 [h·y^-1^] | 8.21 | ± | 5.96 | 11.07 | ± | 6.79 | 0.282 | 0.10 |
| Zone 4 [h·y^-1^] | 28.82 | ± | 6.22 | 32.31 | ± | 8.46 | 0.250 | 0.12 |
| Zone 5 [h·y^-1^] | 19.94 | ± | 5.82 | 23.04 | ± | 9.33 | 0.335 | 0.08 |

**Supplementary Table 2**: Weekly training patterns across Lockdown (L) and Control (C) periods. Data are mean ± SD. *P* value and effect size (ES) statistics represent pairwise comparisons between respective periods (C1 vs. L1, C2 vs. L2).

| **Weekly training patterns** | **C1** | | | **L1** | | | ***P*** | **ES** | **C2** | | | **L2** | | | ***P*** | **ES** |
| --- | --- | --- | --- | --- | --- | --- | --- | --- | --- | --- | --- | --- | --- | --- | --- | --- |
|  | 19/20 | | | 20/21 | | |  |  | 19/20 | | | 20/21 | | |  |  |
| **Total Training:** |  |  |  |  |  |  |  |  |  |  |  |  |  |  |  |  |
| Training Duration [h·wk^-1^] | 11.6 | ± | 2.42 | 19.28 | ± | 3.14 | **< 0.001** | **0.92** | 15.09 | ± | 1.86 | 16.25 | ± | 1.29 | **0.011** | **0.46** |
| **Training Mode:** |  |  |  |  |  |  |  |  |  |  |  |  |  |  |  |  |
| Specific [h·wk^-1^] | 3.53 | ± | 2.93 | 3.59 | ± | 1.87 | 0.933 | 0.00 | 10.85 | ± | 1.16 | 12.24 | ± | 1.34 | **0.002** | **0.61** |
| Non-specific [h·wk^-1^] | 5.12 | ± | 1.63 | 12.14 | ± | 2.61 | **< 0.001** | **0.87** | 2.45 | ± | 1.10 | 2.38 | ± | 0.77 | 0.717 | 0.01 |
| Strength [h·wk^-1^] | 1.41 | ± | 1.28 | 2.81 | ± | 0.84 | **< 0.001** | **0.69** | 1.31 | ± | 0.47 | 1.18 | ± | 0.55 | 0.394 | 0.07 |
| Other [h·wk^-1^] | 1.55 | ± | 1.32 | 0.74 | ± | 1.13 | **0.025** | **0.38** | 0.48 | ± | 0.48 | 0.45 | ± | 0.56 | 0.708 | 0.01 |
| **Intensity Distribution:** |  |  |  |  |  |  |  |  |  |  |  |  |  |  |  |  |
| Zone 1 [h·wk^-1^] | 1.81 | ± | 2.48 | 7.47 | ± | 3.59 | **0.001** | **0.64** | 9.04 | ± | 3.09 | 10.46 | ± | 3.26 | 0.224 | 0.13 |
| Zone 2 [h·wk^-1^] | 3.81 | ± | 3.01 | 1.32 | ± | 2.44 | **0.055** | **0.30** | 2.21 | ± | 2.26 | 2.16 | ± | 3.34 | 0.946 | 0.00 |
| Zone 3 [h·wk^-1^] | 0.01 | ± | 0.03 | 0.04 | ± | 0.10 | 0.409 | 0.06 | 0.02 | ± | 0.04 | 0.07 | ± | 0.09 | 0.097 | 0.23 |
| Zone 4 [h·wk^-1^] | 0.06 | ± | 0.12 | 0.33 | ± | 0.30 | **0.015** | **0.43** | 0.65 | ± | 0.24 | 0.65 | ± | 0.20 | 0.992 | 0.00 |
| Zone 5 [h·wk^-1^] | 0.24 | ± | 0.20 | 0.06 | ± | 0.08 | **0.023** | **0.39** | 0.56 | ± | 0.18 | 0.76 | ± | 0.30 | 0.095 | 0.23 |

**Supplementary Table 3A:** Submaximal incremental test protocol. Athletes performed increments of fixed length (time: t mm:ss) with fixed starting velocities (v: m/s) which could be individually adjusted by a tension cord around athletes‘ hips to match the target Heart Rate zone (HR; % HR_max_).

| **Increment** | **Intensity Zone** | **% HR_max_** | **t _(mm:ss)_** | **Incline _(°, %)_** | **v _(m/s) ♀_** | **v _(m/s) ♂_** |
| --- | --- | --- | --- | --- | --- | --- |
| 1 | I1 | 60-72% | 06:00 | 2° / 3.5% | 2.9 | 3.2 |
| 2 | I2 | 73-82% | 06:00 | 2° / 3.5% | 3.5 | 3.9 |
| 3 | I3 | 83-87% | 06:00 | 2° / 3.5% | 4.1 | 4.3 |
| 4 | I4 | 88-92% | 06:00 | 2° / 3.5% | 4.7 | 5.3 |
| 5 | I5 | 93-97% | 06:00 | 2° / 3.5% | 5.3 | 6.0 |

**Supplementary Table 3B:** Maximal ramp test protocol. After the initial Warm-up athletes performed stages of 1 min length (time: t mm:ss), with fixed increments of 20s at different fixed inclines and velocities (v: m/s). The v was incrementally increased per stage.

| **Stage** | **Incline _(°, %)_** | **t _(mm:ss)_** | **v _(m/s) ♀_** | **v _(m/s) ♂_** |
| --- | --- | --- | --- | --- |
| **Warm-up** | 1° / 1.8% | 01:00 | 4.20 | 4.70 |
|  | 1° / 1.8% | 01:00 | 5.40 | 6.00 |
| **1** | 1° / 1.8% | 00:20 | 5.65 | 6.30 |
|  | 4° / 7.0% | 00:20 | 4.05 | 4.50 |
|  | 7° / 12.3% | 00:20 | 2.60 | 2.90 |
| **Next stage** | 1° / 1.8% | 00:20 | prev. Stage + 0.22 | prev. Stage + 0.25 |
|  | 4° / 7.0% | 00:20 | prev. Stage + 0.18 | prev. Stage + 0.20 |
|  | 7° / 12.3% | 00:20 | prev. Stage + 0.13 | prev. Stage + 0.15 |
